# Infection prevention behaviour and infectious disease modelling: a review of the literature and recommendations for the future

**DOI:** 10.1186/s12889-018-5223-1

**Published:** 2018-03-09

**Authors:** Dale Weston, Katharina Hauck, Richard Amlôt

**Affiliations:** 1 Behavioural Science Team, Emergency Response Department Science & Technology, Public Health England, Porton Down, Salisbury, UK; 2 Department of Infectious Disease Epidemiology, School of Public Health, Imperial College London, London, UK

**Keywords:** Infectious disease, Human behaviour, Mathematical modelling, Literature review, Protective behaviour

## Abstract

**Background:**

Given the importance of person to person transmission in the spread of infectious diseases, it is critically important to ensure that human behaviour with respect to infection prevention is appropriately represented within infectious disease models. This paper presents a large scale scoping review regarding the incorporation of infection prevention behaviour in infectious disease models. The outcomes of this review are contextualised within the psychological literature concerning health behaviour and behaviour change, resulting in a series of key recommendations for the incorporation of human behaviour in future infectious disease models.

**Methods:**

The search strategy focused on terms relating to behaviour, infectious disease and mathematical modelling. The selection criteria were developed iteratively to focus on original research articles that present an infectious disease model with human-human spread, in which individuals’ self-protective health behaviour varied endogenously within the model. Data extracted included: the behaviour that is modelled; how this behaviour is modelled; any theoretical background for the modelling of behaviour, and; any behavioural data used to parameterise the models.

**Results:**

Forty-two papers from an initial total of 2987 were retained for inclusion in the final review. All of these papers were published between 2002 and 2015. Many of the included papers employed a multiple, linked models to incorporate infection prevention behaviour. Both cognitive constructs (e.g., perceived risk) and, to a lesser extent, social constructs (e.g., social norms) were identified in the included papers. However, only five papers made explicit reference to psychological health behaviour change theories. Finally, just under half of the included papers incorporated behavioural data in their modelling.

**Conclusions:**

By contextualising the review outcomes within the psychological literature on health behaviour and behaviour change, three key recommendations for future behavioural modelling are made. First, modellers should consult with the psychological literature on health behaviour/ behaviour change when developing new models. Second, modellers interested in exploring the relationship between behaviour and disease spread should draw on social psychological literature to increase the complexity of the social world represented within infectious disease models. Finally, greater use of context-specific behavioural data (e.g., survey data, observational data) is recommended to parameterise models.

**Electronic supplementary material:**

The online version of this article (10.1186/s12889-018-5223-1) contains supplementary material, which is available to authorized users.

## Background

Research in the field of epidemiology has traditionally employed mathematical models to successfully reproduce the observed incidence and prevalence of diseases [1], including influenza [2], HIV [3], smallpox (e.g., [4]), and malaria (e.g., [5]), amongst others. These models are important both for developing our understanding of potentially novel disease strains (e.g., A/H1N1, [6]), and also for planning responses to infectious disease outbreaks. For example, models incorporating disease control measures (e.g., vaccination, quarantine, school closures) can be used to examine the contexts in which specific interventions are likely to be more or less effective (e.g., [4, 7, 8]).

Given both the critical role of person to person transmission in the spread of outbreaks (e.g., respiratory infections, Ebola) and the importance of behavioural compliance in the success of multiple infection control interventions (i.e., individuals need to consent to vaccination, or adhere to quarantine restrictions), it is vitally important that human behaviour is accurately represented within infectious disease models. However, recent epidemiological research has noted a limitation of traditional mathematical models of disease spread: they often do not allow for heterogeneous behavioural responses within a population (e.g., [9]). This emphasis on homogenous behaviour is broadly inconsistent with what we know about human behaviour from decades of psychological research and theory in the context of health-related behaviour change. For example, a meta-analytic review of research involving the Theory of Planned Behaviour (a psychological theory of behaviour change, e.g., [10]) found that this theory explained 39% of behavioural intentions, with intentions subsequently explaining 27% of actual behaviour [11]. In other words, this well cited theory (implicated in 13% of behaviour change articles, [12]), explains less than half of all individual’s health-related behaviour. It is therefore critical that infectious disease models seeking to incorporate human behaviour do so in a way that realistically reflects its heterogeneous nature.

Before recommendations can be made for how to better operationalise human behaviour in infectious disease models, we need to clearly understand how human behaviour during an infectious disease outbreak is currently modelled. The large scale scoping review presented within this paper represents an attempt to collate and summarise the state of the art concerning the incorporation of behaviour designed to protect oneself against infection within mathematical models of infectious disease spread, for example, vaccination, distancing oneself from other individuals (social distancing), condom use, or hand washing. More specifically, we were interested in developing a detailed understanding of: what diseases and infection prevention behaviours are modelled across the literature; how the behaviour is modelled (with an explicit interest in understanding both the mechanism of modelling and the components that contribute to behaviour change), and; what theoretical background is presented to support the modelling of infection prevention behaviour (if any).

A wide range of literature drawn from the behavioural sciences is available to assist modellers in developing more realistic models of human behavioural responses to infectious disease outbreaks. For instance, Susan Michie and colleagues worked with health behaviour experts (health psychology theorists, health psychologists, and health services researchers) to reach a consensus on 12 domains (later revised to 14 [13]) that are central to the explanation of behaviour change [14]. Furthermore, recent research within social psychology has suggested an important role for social relationships in informing individuals’ health-related behaviour (‘The Social Cure’ e.g., [15]). The outcomes of this review are presented and discussed in the context of this available literature, resulting in a series of recommendations designed to help infectious disease modellers to model human behaviour by incorporating insights from the behavioural sciences.

## Methods

In order to ensure a transparent and systematic approach to our scoping review, we developed our search strategy, inclusion/ exclusion criteria, and data extraction process based on Arksey & O’Malley’s scoping review framework [16]. We opted to use the scoping study methodology rather than a systematic review methodology as we were not concerned with systematically assessing the quality of all available literature; a task that would befit the explicit use of a systematic review approach. Instead, we were focused on: a) mapping and collating the existing literature to identify current best practice for incorporating human behaviour into infectious disease models, and; b) identifying aspects of human behaviour modelling that could be improved through the incorporation of insights from both health and social psychology. Both of these aims are consistent with the use of the scoping review methodology as described in the literature [16, 17].

## Identifying relevant studies

As per Arksey and O’Malley’s [16] recommendation, our search strategy was designed to be as comprehensive and inclusive as possible in the first instance. The search strategy contained terms relating to behaviour, infectious disease, and mathematical modelling. All terms were initially developed by the first author. In the case of the mathematical modelling terms, the first author identified and extracted commonly occurring modelling methods/ keywords presented in the modelling literature. These terms were then reviewed by infectious disease modelling colleagues at Public Health England and Imperial College London to identify any obvious missing terms. All terms were ultimately discussed and agreed with by all members of the primary research team.

The first implementation of the search strategy (run on PubMed on 3/7/2015) yielded only 75 records (see Results section), indicating problems with the optimisation of the strategy. Iterative development of the search strategy ultimately yielded the final, optimised search strategy. This strategy included title/ abstract keyword searches and thesaurus database terms.^1^ The number of papers identified was limited in the first instance by using specific, yet ‘unexploded’ thesaurus terms to try and maximise the number of relevant included papers while minimising irrelevant results. Time and resource constraints also precluded backward and forward citation searching within included papers.

Final searches were conducted on both Medline (on 29/7/2015) and Embase (on 29/9/2015) in the first instance, using the Healthcare Database Advanced Search (HDAS). A further ‘top up’ search was optimised and run on PubMed (on 2/10/2015) to capture recently published papers that had not yet been indexed by Medline. The search strategy employed on Medline is presented in the Additional file 1. Given the nature of this review, the PICOS (Participants, Interventions, Comparisons, Outcomes, and Study Design) criteria detailed in the PRISMA (Preferred Reporting Items for Systematic Reviews and Meta-Analyses) 2009 checklist [18] was deemed inappropriate for designing the search strategy. For instance, we did not anticipate the articles would include research participants or any kind of intervention.

## Selection criteria

The inclusion/exclusion criteria employed during our review were initially developed, in conjunction with our search strategy, to be as inclusive as possible. The a-priori selection criteria simply specified that papers would be included if they presented a mathematical model pertaining to the transmission of an infectious disease within a population, with a particular emphasis on models that present heterogeneous behaviour by agents. As per the scoping review framework, our study selection criteria developed as a function of increasing familiarity with the literature [16]. In this way we were able to both: a) narrow the focus of the review, and; b) reduce uncertainty in the selection process. For instance, we initially proposed to include papers identified within review articles as well as grey literature (i.e., non-peer reviewed papers), but refined the criteria to exclude these given the breadth of the identified peer-reviewed literature.

The final inclusion/exclusion criteria are presented in Table 1. In order to ensure a clear focus for the review, the authors agreed to focus on original research articles that present a mathematical model of human-to-human infectious disease spread, in which individual’s self-protective behaviour varied endogenously (i.e., within the model) rather than as a function of specific modeller-imposed behavioural interventions. The decision to exclude models in which behaviour is exogenously determined (i.e., through modellers modifying a single parameter value) was taken in order to ensure that our review focused on best practice for modelling human behaviour. For the purpose of this review, parental decision-making (e.g., with respect to vaccination) was considered to be self-protective behaviour as the parents are making the decision on behalf of the child, who is legally unable to make the decision themselves; the parent therefore represents a proxy for the child’s own decision making. Furthermore, we chose to exclude papers that used only statistical/ econometric models with no mathematical transmission modelling as this practice is commonplace within psychology (e.g., [19]) and so does not merit further assessment. No limits were placed on the publication date of included articles. These criteria substantially build upon those employed in an earlier review of the same topic (specifically, the emphasis on individual, endogenous decision making) [20]. Furthermore, although developed and implemented independently of one another, our criteria share a common impetus with the criteria developed and employed in another recent review of human behaviour within infectious disease models [21]. Considered together, these criteria therefore allowed us to ensure, as far as possible, that the mechanisms for modelling human behaviour reviewed herein reflect attempts to consider the kind of complex, individual processes underlying human behaviour identified in the psychological literature (e.g., [14]).

**Table 1:** Final inclusion/exclusion criteria used for record screening

Include	Exclude
Include only articles that focus on human to human transmission	Exclude all articles that do not focus on human to human transmission (e.g., vector-borne)
Include articles with a focus on *self-*prevention behaviour (i.e., preventing one’s own infection) in response to an outbreak/epidemic (including parental decision making)	Exclude articles that focus on behaviour concerned with infection risk in *other-*individuals (i.e., preventing others from being infected, possibly by the self)
Include articles that endogenously model behaviour^a^	Exclude articles concerned with exogenous behaviour change (e.g., an intervention) unless the behaviour is mediated by other factors that change/respond within the model (e.g., age, risk preferences, etc.)
Include articles that incorporate individual level behavioural elements (e.g., decision making)	Exclude ‘population models’ in which behaviour is universal (i.e., determined by a specific exogenous parameter) within a given group.
Include articles employing a mathematical transmission model or modelling component to represent behaviour change	Exclude articles using only statistical/econometric models (e.g., regression analyses, and related approaches)
Include articles that are published in peer reviewed academic journals	Exclude conference proceedings, posters, grey literature.
Include articles presenting a novel mathematical model	Exclude review articles
Include only articles written in the English language	Exclude all non-English language articles

^a^Simply including a single static parameter value for behaviour change (e.g., a wholesale change from X to Y transmission rate or a static rate of behaviour change of X% when prevalence reaches a certain threshold value or an intervention is implemented) employed uniformly was not counted as endogenous behaviour change in the model

### Charting the data

All papers that were selected for inclusion in the review were subjected to a standardised data extraction procedure that was developed by the first author in the first instance, and was agreed by all other authors. This procedure was revised and extended twice: once during an interim presentation of the review outcomes to a team of infectious disease modellers and behavioural economists on 29th February 2016, and once during a Public Involvement workshop on 30th September 2016. Ultimately, the following information was extracted from all papers: authors; date of publication; the type of model used; the disease that is modelled; the behaviour that is modelled; how this behaviour is modelled; whether and how information or awareness spread is modelled; whether and how fading or decaying memory is modelled; whether and what theoretical background for behaviour change is provided; whether and what comparisons there were between the model that incorporates endogenous human behaviour and a control model (i.e., a model which either does not incorporate endogenous behaviour, or does not incorporate behaviour at all); whether the model was parameterised or fitted to data (and if so, which data sources); and the main conclusions concerning the impact of behaviour on model outcomes (e.g., the size of the epidemic). The extraction criteria are presented in their entirety in the Additional file 1. In the interests of brevity, we have focused on the selection of the data that pertains to our central research questions in the main results section (i.e., data relating to modelling mechanisms, behavioural constructs, and theoretical underpinnings). All other extracted data is available on request from the first author.

## Results

### Study selection

**Fig. 1:**
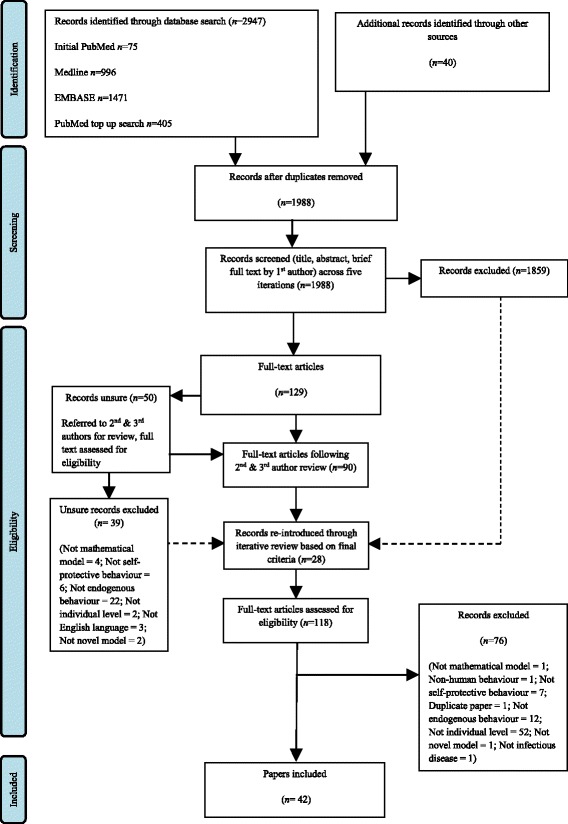
PRISMA diagram detailing the stages of the review process (adapted from [18]). Note that as this was a scoping review, an iterative process was followed (both within the ‘records screened’ stage and subsequent dashed lines) to identify papers for inclusion in the final review

The revised search strategy run on Medline and Embase with a ‘top up’ search run on PubMed revealed 2872 records. When combined with the 75 papers resulting from the initial PubMed search and the 40 additional records identified through other sources (i.e., literature identified when developing the rationale and protocol for the review), this yielded an initial total of 2987 records. Following the removal of duplicates, 1988 records were retained for title, abstract, and brief full text (in the case of papers about which the first author was uncertain) screening. A PRISMA diagram summarising the broad stages of the screening process is presented in Fig. 1.

At this stage, the screening process became iterative as the criteria developed and became more exclusive. In the first case, the first author screened the title and abstract for all remaining papers, sorting these into ‘include’, ‘exclude’, and ‘unsure’ for each database search (iteration one). The ‘unsure’ papers were then subjected to re-assessment (with brief full text examination if/when required; iteration two), followed by re-assessment of the papers included in both iteration one and iteration two (iteration three) to ensure that all included papers met the refined exclusion criteria. Any papers that remained ‘unsure’ were further screened by the first author (title/abstract or brief full text where appropriate; iterations four and five). In total, 129 papers were retained for full text assessment following all five iterations (including 79 papers for full-text review and 50 papers still ‘unsure’), with 1859 papers excluded.

The 50 remaining unsure papers were both reviewed by the first author, and were referred to the second and third authors for review. Of these 50 papers, 39 were excluded at this stage, leaving 90 papers for full-text assessment. Following this review process, the exclusion criteria were refined for the final time and were applied by the first author to all papers previously excluded during iteration 2 onwards. This revealed 28 formerly excluded papers that were re-included for full text analysis. Overall there were 118 papers (90 following unsure paper assessment plus 28 formerly excluded papers) subjected to thorough full-text assessment. Following this final, full-text assessment 42 papers were retained and included in the qualitative synthesis.

### Notable inclusion/exclusion decisions

Despite applying our stringent inclusion/exclusion criteria in an objective and systematic fashion, there is, unavoidably, some subjectivity involved in paper selection. In an attempt to make this subjectivity transparent, this section will highlight some of the inclusion/exclusion decisions made.

As mentioned in the Introduction, human behaviour is not straightforward to predict. We therefore opted to focus on papers that included complex, individual decision making with regard to the adoption or avoidance of behaviour. A good example of such decision making processes involves a model in which susceptible agents calculate the cost and benefits of both risk-taking and protective behaviours by drawing a random sample of model agents, in conjunction with their previous estimates, to draw inferences about disease prevalence and inform their decision making [22]. Similarly, Fenichel et al. [23] model social distancing decisions as a function of an agent’s current-period utility, which depends on their health state and their interaction with other individuals. In other words, an individual’s decision making is based on a cost-benefit calculation that is unique to that agent (i.e., by considering their specific social contacts, e.g., [24], or comparison to an agent drawn randomly from the model population, e.g., [25]).

Although these models represent a ‘gold standard’ for inclusion in this review, there are also models that consider individuals’ decision making, but based on population level values. For example, whereas the ‘gold standard’ models may incorporate cost-benefit calculations that are dependent upon characteristics specific to the individual (e.g., behaviour among their own social network), there are models that incorporate cost-benefit calculations in which, for example, susceptible agents choose a public activity level that is based on their knowledge of overall prevalence and public behaviour [26]. Similarly, Bhattacharyya and Bauch [27] present a model of vaccination in which the payoff for an individual vaccinating in a given week is based on the average vaccine coverage across the entire population. Overall, papers of this nature were included as they represent the complex nature of behavioural decision making, albeit with some more population level input.

There were also additional included papers that met the individual level behaviour requirement, but that did not explicitly include the elements of decision making discussed above. For example, models in which individuals evaluate the number of behavioural adopters/infected individuals among their contacts and modify their behaviour once an individualised threshold number of contacts drawn from a behavioural survey is met [28, 29]. This is in contrast to models, excluded as population level, in which individuals behaviour is based on the general, modeller-defined proportion of their contacts who are engaged in that behaviour (e.g., [30, 31]).

Finally, awareness/ behaviour may spread within a model through person to person contact (e.g., [32, 33]), this relies on a modeller-defined global rate of contact and thus papers of this nature were excluded. Funk, Gilad, Watkins, and Jansen [34] instead present a more individual-level model of awareness spreading. In this model, an individual’s level of awareness (and consequent likelihood of infection) is determined by the number of individuals that the information passed through prior to them [34]. It therefore represents a more individual level representation of behaviour than the standard contact – infection models described above.

As should be evident from the above, the line between inclusion and exclusion can be broadly considered in relation to the modellers’ attempt to accurately represent the complex and multifaceted nature of individual human behaviour. The papers presented in the remainder of this review represent, in the authors view, the best attempts at incorporating human behaviour that are consistent with our stringent inclusion/ exclusion criteria.

### Study characteristics

As detailed previously, 42 papers were retained following full text analysis [22–29, 34–67]. The full citation list of included papers is included in the Additional file 1. In this section we present data relating to the date of publication and discuss aspects of the model design used within the included papers (e.g., the types of model, behaviour, and disease represented within the included papers). The data discussed through the rest of this Results section is presented in the Additional file 2. The full extracted data is available on request from the first author.

#### Date of publication

**Fig. 2:**
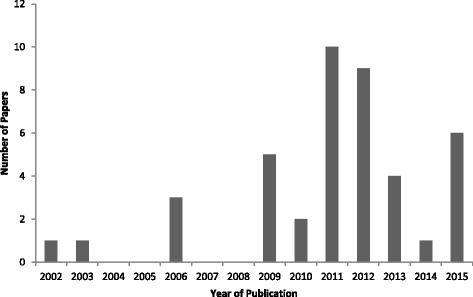
Number of papers presenting infectious disease transmission models with endogenous behaviour change, by year of publication

All included papers were published since 2002 with a broadly upwards trend until a peak in 2011 (10 papers [25–28, 37, 42, 45, 50, 58, 66]), with a subsequent 40% decline in papers published by 2015 (6 papers [35, 46–49, 54], Fig. 2). The 2011 (and to a lesser extent 2012) peak corresponds with the aftermath of the A/H1N1 2009 pandemic, which ended in August 2010 [68]. Indeed, of the 19 included papers published in 2011–2012, seven explicitly concerned influenza (seasonal or epidemic) or an influenza-like infection [25, 28, 29, 37, 42, 58, 65]. This data suggests that the inclusion of individual health-protective human behaviour within models of infectious disease spread is a relatively recent development, which may be related to the influenza A/H1N1 pandemic.

#### Country of origin

To give an indication of the geographical spread of this modelling work, we extracted data concerning the country in which the first author’s institution was based. Over half of all included papers originated from the USA (23 of 42 papers [22, 23, 25, 26, 28, 29, 37, 39–42, 44–47, 49, 52–54, 61–64]), with Canada (11 [27, 35, 36, 38, 48, 51, 55–57, 65, 66]), Italy (5 [43, 50, 58–60]), China (2 [24, 67]), and the UK (1 [34]) making up the full cohort of nations in which the first author was based when the work was published.

#### Disease modelled

The majority of the models described in the included papers did not specify the disease to which they related (22 of 42 papers [22–24, 26, 27, 34, 36, 41, 43, 45, 50–53, 57, 59–64, 66]). Where models did relate to a specific disease, this was most commonly influenza or an influenza-like-infection [25, 28, 29, 35, 37, 42, 46, 48, 58, 65, 67]. This focus on influenza is consistent with the peak publication date occurring in the aftermath of the 2009 UK A/H1N1 pandemic.

#### What behaviour is modelled

The majority of papers focused on vaccination (17 cases [22, 24, 25, 27, 38, 40, 42, 43, 51, 52, 54–57, 65, 66]) or social distancing behaviour (12 cases [23, 26, 41, 45, 46, 50, 53, 59, 60, 61, 63, 64]). A smaller, yet substantial proportion of cases either modelled one or more general behavioural responses (10 cases [28, 29, 35, 37, 39, 47–49, 58, 67]) or did not specify the behaviour that was being modelled (1 case [34]).^2^ There was, therefore, very little variation in the type and nature of self-protective behaviour presented in the included papers.

#### Type of model

A range of different methods were employed across the included papers, with some papers either detailing multiple, combined models, or incorporating components from different modelling methods. It is therefore difficult to precisely quantify the specific models that were most commonly used across all papers. There are, however, some broad conceptual or methodological similarities that can be outlined. First, the majority of these papers employed a compartmental model (e.g., a Susceptible-Infectious-Recovered [SIR] model) to represent disease spread (e.g., [23–27, 34, 36, 40, 41, 43, 45, 47, 54–56, 58–63, 67]). Second, network modelling components (i.e., behaviour and/ or disease spread represented on a social network) were employed across multiple papers (e.g., [29, 35, 37, 42, 46, 49, 51–53, 56, 57, 65, 67]) as, to a lesser extent, were Agent Based Modelling approaches (a computational modelling approach in which agents are individual, autonomous decision-makers [69], (e.g., [24, 25, 28, 35, 44, 46, 48, 49]), both to model behaviour and disease spread. Third, a substantial proportion of the papers explicitly incorporated economic or game theoretic elements within their infectious disease models (i.e., to model individual behavioural decision making) (e.g., [22, 24, 27, 35, 38, 47, 51, 54, 61, 62, 67]). Finally, a common approach to modelling the spread of individual protective behaviour during an infectious disease outbreak was to include more than one model in the analysis [24–27, 35, 37, 46, 47, 53–55, 61, 62, 65–67]; for instance, a behavioural model (e.g., economic games, individual decision making) linked with an infectious disease transmission models (e.g., SIR model) within the paper (e.g., [24, 26, 27, 47, 61]).

### Synthesis of results – Infectious disease modelling of human behaviour

As the nature of our review precluded the extraction of detailed PICOS related information, an extensive discussion of comparisons and outcomes was not appropriate. Moreover, the relatively large number of papers included in this review precludes an in depth assessment of the results from all individual studies. Our analysis instead focused on a summary and synthesis of the extracted data related explicitly to the modelling of human behaviour.

#### How the behaviour is modelled

During data extraction, extensive information concerning the method of modelling human behaviour was collated. In all bar six [28, 29, 34, 37, 46, 49] of the 42 included papers, behaviour was modelled using either a cost-benefit calculation [22, 23, 26, 27, 35, 36, 39–42, 44, 45, 47, 48, 50, 51, 53, 56, 57, 60, 61, 63, 65], behavioural imitation [64], or an integration of the two [24, 25, 38, 43, 52, 54, 55, 58, 59, 62, 66, 67]. Typically, the cost-benefit calculation involves agents considering the payoff of comparing the utilities associated with engaging in protective behaviour against the utilities associated with remaining susceptible (e.g., [22, 41, 42, 57, 62, 65]). Some components of these cost-benefit calculations may be static parameters, however they can also be variable; for example, in Durham and Casman’s [44] model, perceived benefits of health protective behaviours are modelled as a static, unchanging value, whilst perceived susceptibility to disease is influenced by recent disease prevalence within the model. These prevalence-based utilities can be based on information from a single modelled infection season (e.g., [37, 38]), or a combination of current and past modelled seasons (e.g., [26, 44, 58]). For example, whole model or local contact infection prevalence can influence the risk of infection (e.g., [22, 38, 40, 43, 44, 50]), or; the payoff of a protective strategy can be proportional to the number of individuals engaging in that strategy (e.g., [54]).

The method of incorporating behavioural imitation varied across the models, but commonly involved either a prevalence-based mechanism (e.g., adoption of the most prevalent behavioural strategy within the model, e.g., [62, 66]) or the random selection of another individual (either randomly from the entire model, e.g., [25, 55]; or from within one’s contact network, e.g., [24, 52, 67]) and comparison of the sampled strategy against one’s own. All bar one of the 13 models that incorporated behavioural imitation also incorporated a mechanism of cost-benefit calculation [64]. This either involved incorporating two distinct strategies—one for imitation and one for cost-benefit calculation (for example, with the distribution of strategy within the model determined by a static parameter [52])—or the incorporation of both imitation and cost-benefit calculation together. For example, individuals may select another individual within their model (i.e., randomly from the entire model or from the individual’s immediate neighbours) and then compare the payoffs of their relative behavioural strategies – imitation occurs when the target individual’s payoff is greater than one’s own (e.g., [24, 25, 54, 55, 58, 59, 67]). The final remaining imitation strategy (that did not incorporate both cost-benefit calculations and behavioural imitation) models a situation in which individuals can observe the health status of others and are more likely to adopt the behaviour of a healthy person than of an unhealthy person (regardless of whether this behaviour is careful or risky) [64].

The remaining six models used a range of different strategies for modelling behaviour, including: information-dependent disease transmission that varies as a function of the number of individuals the information has previously travelled through [34]; behavioural strategies dependent upon population class (e.g., socio-economic group, age [37]); behaviour dependent upon the individual agent’s parameter-determined cognition (e.g., related to media-reported illness attack rates, individual’s social network degree [46]); behaviour dependent upon number of infected individuals or number of individuals that adopt the protective behaviour reaching an individualised threshold [28, 29], and the use of random Bernoulli trials to govern whether individuals cooperate with public health interventions or not [49].

#### Behavioural constructs modelled

Further exploration of the data presented in the included papers was conducted in order to identify the key constructs that contributed to the modelling of human behaviour. This is not intended as an exhaustive list of all constructs that may contribute to behaviour, but instead represents the constructs that were identified as most central to the modelling of human behaviour within each paper. Similarly, the example references are provided below to highlight each construct, this is not an exhaustive list of all identified papers employing these constructs; more detail can be found in the Additional file 2.

##### Cognitive constructs

These are the most commonly applied constructs within the included literature, and are most typically incorporated as part of the cost-benefit calculation models discussed above. These constructs focus generally on the costs and benefits of remaining susceptible and/ or engaging in protective behaviour. For instance: risk (likelihood) of infection (this can be based on prevalence either within one’s contacts or the whole model population, and on either the current infection season or a cumulative memory across past seasons, e.g., [28, 29, 42, 56–58]), costs associated with infection (e.g., loss of health, life expectancy, [52, 65]), costs associated with self-protection (e.g., side effects, monetary cost, time cost, e.g., [24, 25, 43, 65]), vaccine efficacy (e.g., [65]), costs associated with antisocial behaviour (for social distancing, e.g., [61]).

##### Social constructs

Although perceived risk of infection as determined by prevalence is listed above as a cognitive construct, the emphasis on considering the health status of other individuals can also constitute a social construct; particularly in the cases where estimates of prevalence are based upon an agent’s immediate social group or context (i.e., their neighbours) rather than whole-population prevalence (e.g., [24, 52, 67]. The social implications of one’s own behaviour are also modelled; for example, individuals decision to engage in protective behaviour (e.g., vaccination) can be influenced by the perception that failing to modify ones behaviour will result in harm for others, or that modifying one’s behaviour may improve the health of other individuals (e.g., [35, 62]). Some of the included papers also include social norm proxies within their models. First, individuals may identify the number of behavioural adopters within their contacts and modify their own behaviour if this proportion reaches a given threshold [28, 29]. Second, social norms are also represented as a modification to the payoffs for engaging in protective behaviour, that is, the payoff for engaging in a protective behaviour varies as a function of the number of individuals within the population that are also engaging in that behaviour [54, 55].

Behavioural comparison and imitation represent one further key social construct in the modelling of human behaviour. As outlined above, this can involve comparison with another individual randomly selected from the entire population, e.g., [25, 55], or it can be restricted to comparisons within one’s immediate social environment, e.g., [24, 52, 67]. Similarly, the transmission of information, cultural traits, or awareness from person to person can also constitute a social construct; for example, when this transmission involves an awareness of how many contacts information has passed through [34], or trait transmission as a function of perceived health status of a contact [64].

##### Other constructs

The following additional constructs also contributed to the modelling of health-protective behaviour in the included papers: demographic information (e.g., age, socio-economic status, family status, [37, 63]) external information concerning an outbreak (e.g., media coverage, [44, 46]), and; local temperature [46]).

#### Background literature concerning behaviour

**Fig. 3:**
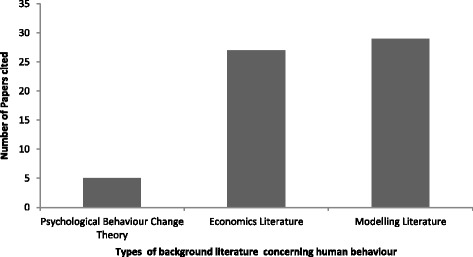
The number of papers included within the review (*n* = 42) that cite: a) psychological behaviour change theory; b) economics literature relating to human behaviour, and/ or; c) previous mathematical models that have incorporated human behaviour. Note: individual papers included in the review may have incorporated more than one of these categories of literature and so may be numerically represented multiple times

Every paper included in our analysis presented background literature to support the modelling of protective behaviour. As our primary concern is to explore the extent to which psychological constructs and theories have been incorporated into infectious disease models, all papers were examined to determine whether they cited psychological health behaviour theories (e.g., [12]). Close examination of the papers revealed two broad additional classifications of background literature related to human behaviour: economic literature (e.g., game theory), and previous models that have incorporated human behaviour. All bar two of the included papers [28, 37] contained literature that fit one or more of these criteria; this indicates that we are unlikely to have missed a substantial literature when developing our classification. The number of papers containing each of the three classifications of behaviour change literature is presented in Fig. 3.^3^

Examination of Fig. 3 reveals that although the majority of papers do include either economics literature concerning behaviour or previous modelling literature that incorporates human behaviour (or both), only five of the 42 included papers make explicit reference to a well-recognised psychological theory of health behaviour change (see [12]) [44, 46, 48, 54, 55]. These five papers cite a range of different theories (including one review of cognitive behaviour theories generally [46], the Theory of Planned Behaviour (TPB) [44], Theory of Reasoned Action (TRA) [44], Prospect Theory (PT) [54], and the Transtheoretical Model (TTM) [44]). However, the most commonly cited theory of behaviour change (cited by four of the five identified papers [44, 48, 54, 55]) is the Health Belief Model (HBM).

#### Behavioural data used – Parameterisation and fit

In the first instance we were simply interested in whether the models detailed in our included papers were applied to, or parameterised by existing data, and if so, which data. In the first instance, 26 papers made reference to either previous literature or data in the parameterisation or validation (fit) of their models [23, 25, 27–29, 35–38, 42–46, 48–50, 53–59, 63, 65]. Of these papers, 21 explicitly use data sources within the paper (excluding reference to academic papers) [28, 29, 35–38, 42–44, 46, 48–50, 53–58, 63, 65]; our examination of these papers yielded three broad classifications of data sources. First, survey response data is used to accurately model human behaviour; for example, two notable examples make use of a behavioural survey (in which participants were asked to list the number of friends from a maximum of 10 that would have to be vaccinated in order for the respondent to consider vaccination) to develop individualised social thresholds for behaviour adoption [28, 29]. Second, demographic (e.g., census) data is used to model travel behaviour; for example, in [50], flight data is used to model travel. Similarly, in [37], survey responses were used to determine household activity. Third, epidemiological data concerning vaccine uptake is used; for example, UK pertussis vaccine coverage data [55], and ICONA working group data concerning Italian MMR vaccine uptake data from 1996 to 2008 [43].

## Discussion

The application of our detailed search strategy across three electronic article databases returned 42 papers focused on the incorporation of endogenous human self-protective behaviour within infectious disease models that met our stringent inclusion/ exclusion criteria. All of these papers were published between 2002 and 2015, with a spike in 2011/ 12, with a clear Western (more explicitly, North American) bias in the country of origin; only eight of the papers included in this review originated from outside of North America, with only two of these eight papers originating from outside Western Europe (both China). The majority of the included papers did not focus on a specific disease, although the most commonly modelled was influenza. Consistent with the disease focus, the most commonly modelled protective behaviour was also influenza-related (vaccination). Our included papers therefore seem to reflect a relatively recent increase in infectious disease models incorporating endogenous human protective behaviour that is likely related to the A/H1N1 pandemic.

The broad range of models employed in the included papers precludes any firm conclusions regarding best practice for modelling both human behaviour and infectious disease spread. Nevertheless, it was clear from our data extraction that a substantial proportion of papers employed a dual-model method; using compartmental models (e.g., Susceptible-Infectious-Recovered, Susceptible-Infectious-Susceptible models depending on the disease) to represent disease spread, and economic-style models/games (e.g., cost-benefit calculations) to represent behavioural decision making. Moreover, reflecting the importance of social considerations in the modelling of infectious disease spread, a number of models employed social modelling components (e.g., contact networks; behavioural imitation) to represent the spread of disease or human behaviour.

A range of different cognitive and social constructs (with an emphasis on the cognitive) contributed to the modelling of human behaviour across papers. The cognitive constructs typically focused on the relative costs and benefits of remaining susceptible or engaging in protective behaviour. These included: perceived or actual risk (i.e., of infection or vaccine complications), the social costs of protective behaviour/ benefits of remaining susceptible, and the health costs of remaining susceptible. On the other hand, the social constructs typically focused on either behavioural comparison/ imitation of others within the model, the social consequences of engaging in protective behaviour, or the normative acceptability of engaging in a given behaviour. Very few of the included papers made explicit reference to psychological health behaviour theories when discussing human behaviour, relying instead upon literature from behavioural economics and infectious disease modelling.

Finally, just under half of the papers included in our review made reference to behavioural data in their modelling. Among these papers, there were instances of more thorough incorporation of behavioural data, such as population surveys of vaccination acceptability/uptake and travel behaviour, within the included papers.

Through synthesising the outcomes of our review with the psychological behaviour change and health protection literatures, we develop three central recommendations for how modellers can ensure that human behaviour is incorporated in their infectious disease models in a realistic and representative fashion: The role of psychological theory; the importance of the social world, and the use of behavioural data.

### The role of psychological theory in identifying predictors of human behaviour

Broadly speaking, the emphasis on cognitive components within the included papers corresponds well with the psychological literature on health behaviour change. For example, the Integrative Model of Behavioural Prediction includes behavioural belief, perceived risk, normative belief, and efficacy belief components [70]. Similarly, the emphasis on cost-benefit calculations for engaging in protective behaviour is a feature of multiple psychological models of health behaviour change (for example, the HBM [71]; Protection Motivation Theory (PMT) [72, 73], and; the Extended Parallel Processing Model (EPPM) [74]). The social constructs identified in our review (particularly behavioural imitation and social norms) are also represented in several psychological theories of behaviour change (e.g., Social Cognitive Theory [75]; Social Learning Theory [76]; Theory of Planned Behaviour [10], and; the Integrative Model [70]). However, as previously noted, very few of the included papers made explicit reference to these theories. As a point of reference, recent work has identified 83 theories of behaviour change from across the social sciences [12]. Furthermore, although there is overlap in the constructs used [12], different models have been designed to reflect contextually-specific predictors of behaviour. For example, the HBM (cited most commonly by papers included in this review [44, 48, 54, 55]) was designed to help understand the predictors of preventative behaviour in responses to a health threat [12], thus making it thoroughly appropriate for application within the current context. However, both PMT and EPPM were also designed to help understand predictors of behaviour in this context, but with a particular focus on emotional responses (i.e., fear [12, 72–74]). There is, therefore, a wealth of theoretical literature concerning predictors of behaviour and behaviour change within the social sciences that could be drawn upon to inform the modelling of self-protective health behaviour.

Two papers cited within the current review provide an excellent example of how infectious disease transmission modelling can incorporate a more nuanced representation of human behavioural decision making. Specifically, these models combine statistical modelling (specifically logistic regression modelling based on a combination of previous literature and survey data) with agent-based modelling techniques, to present detailed models of infectious disease transmission that incorporate the HBM [44, 48]. However, there is an inevitable compromise between striving for a realistic presentation of human behaviour, and the requirement and constraints of modelling [44]. Thus, despite the appeal of a nuanced psychological modelling as presented in these examples, we accept that this is not always appropriate or desirable.

The theoretical literature relating to behaviour change may instead be more useful for identifying key predictors of human behaviour that have been overlooked within infectious disease modelling. Indeed, a recent paper posits that an awareness of the main factors underlying human behaviour within psychological models may be sufficient for modelling infectious disease transmission (although the authors do acknowledge the importance of further exploring this issue [77]). By way of an example, consider the role of emotional responding within both PMT and EPPM [72–74]. The role of emotions as a theoretical domain associated with behaviour change [13], and the relationship between emotional responses (e.g., anxiety) and behaviour change within the context of the H1N1 pandemic [78], mark emotional responding as a potentially important predictor of behavioural responses to an infectious disease outbreak. However, no articles included in our review made explicit reference to the modelling of any emotional responses to an infectious disease outbreak. One paper that fell just short of our inclusion criteria did include fear-based responding within a model, and found that relatively low levels of fear-related flight can influence the spread of an infectious disease [32]. In other words, although it is not necessarily prudent to consistently model complex behaviour change theories in their entirety, an awareness of and familiarity with the extensive theoretical literature on health behaviour change could help infectious disease modellers to examine the efficacy of previously understudied predictors of human behaviour within future infectious disease models.

Our first recommendation is, therefore, that infectious disease modellers should draw upon the extensive psychological literature concerning the predictors of health behaviour change when incorporating human behaviour into their models. Although the explicit modelling of complex behaviour change models in their entirety may represent a gold standard for infectious disease modelling (see [44, 48]), this may not always be appropriate [77]. Instead, we recommend that modellers familiarise themselves with the behaviour change literature to both: a) identify previously understudied predictors of self-protective health behaviour, and; b) test the effect of incorporating these predictors into future infectious disease models on model validity. Indeed, the importance of cross-disciplinary work to inform future infectious disease modelling has recently been highlighted within the literature [77]. Recent work by Susan Michie and colleagues to review the behaviour change literature represents an excellent starting point for this endeavour [12–14].

### The importance of social constructs for modelling infection prevention behaviour

Several of the included papers make a clear attempt to incorporate complex social constructs (e.g., contact, imitation, norms) into their modelling of human behaviour. However, as for traditional psychological theories of health behaviour change, this involvement is at a relatively surface level; more could certainly be done to improve the inclusion of social constructs in the modelling of human behaviour. There is a longstanding tradition of research within social psychology that tells us that individuals can be members of a wide range of social/cultural groups that are more or less important to them depending upon the context that they are in (i.e., if you are at work you may identify yourself most strongly according to your profession, whereas if you are watching a football match you may identify yourself most strongly according to the team that you support). These ideas are conceptualised formally as part of Social Identity Theory and Self Categorisation Theory (e.g., [79–82], see also [83]). More recent research in this tradition (such as that presented above) has focused on applying these theories within the context of health behaviour (‘The Social Cure’, see [15]), and it is this literature that is of particular relevance for infectious disease modellers.

By way of example, some of the papers included in this review do incorporate social norms for behavioural uptake (based on either the behavioural uptake of an individual’s contacts or population wide behavioural incidence, e.g., [28, 29, 54, 55]). However, we know from the literature that ensuring the relevance of social norms and recommended health behaviours to one’s salient social group is important for behavioural uptake (e.g., [84–86]). For example, a study of British University students found that participants were more likely to engage in health promoting behaviour (e.g., reduced alcohol consumption) to the extent that they saw themselves as British (a comparatively healthy social grouping) rather than as a University student (a comparatively unhealthy social grouping [85]).

Similarly, behavioural imitation typically occurs within the included papers as a function of behavioural prevalence (e.g., the most adopted behaviour across the model, e.g., [62]) or by comparing one’s own behaviour to the behaviour of a randomly selected other (either drawn from one’s contacts [24] or from the model as a whole [25]). However, the decision to imitate the behaviour of another individual is likely to be contingent upon social group processes. Specifically, both the degree to which one identifies with the social group that this other individual represents within a given context, and the extent to which the other individual is valued (and so has greater influence, i.e., leaders) or devalued (and so has less influence, i.e., deviants) within this group [87]. Based on the above, the impact of social norms and behavioural comparison/ imitation is likely to vary as a function of: a) the group that individuals see as important to them in that context; b) the group membership of the individuals within both their contact network and the population as a whole who have adopted (or recommend) a given behaviour, and; c) the extent to which these other individuals are influential within a given group.

A recent paper outlining recommendations regarding the incorporation behavioural dynamics into infectious disease models has indicated the need to better understand the mechanisms underlying the relationship between behaviour and infectious disease dynamics. Specifically, the authors ask “To what extent do people themselves, their social “networks”, media opinion leaders, or health care providers affect individual behaviour?” [77], p.25). We suggest that insights from the literature outlined in this section could help to develop models designed to answer this question. One method of achieving this could include more detailed stratification of social grouping (with a consideration of the relative importance of different groups) within a modelled population. Surveys containing questions that ask individuals to list social groups that are important to them may be one method of obtaining a more accurate understanding of the distribution (and importance) of social groups within a given population to help parameterise these models.

As previously discussed, we are aware of the tensions between theoretical fidelity and the need for model simplicity [77]. It is, nevertheless, important to ensure that models are sufficiently realistic with regard to social and epidemiological processes to allow for the accurate exploration of potential control policies [4]. For example, the assumption of homogenous or random mixing may be inappropriate for diseases that are transmitted via close contact [4]. To resolve this tension, we extend a recommendation made by Funk and colleagues when considering the extent to which behaviour should be modelled explicitly [77]. That is, we recommend that modellers interested in exploring the interplay between behaviour and disease dynamics should develop a range of models into which social constructs of varying complexity are incorporated, with the resulting outputs compared. As with the previous recommendation, this endeavour is consistent with the importance of cross disciplinary dialogue for developing future models [77], and literature relating to the ‘Social Cure’ [15] would be our recommended starting point.

### The use of behavioural data

Over half of all of the included papers made explicit reference to data concerning human behaviour in the development of their infectious disease models. Of particular interest are the papers that made use of in-depth data sources to inform the modelling of human behaviour, including: epidemiological data concerning vaccine uptake (e.g., [38, 43]); travel survey data (e.g., [28]); census data (e.g., [53]), and; health behaviour surveys (e.g., [28, 44, 48]). By using this detailed behavioural data, modellers can help to ensure the realism of their assumptions concerning human responses to an infectious disease outbreak. Our third recommendation is for modellers to ensure that the presentation of human behaviour within infectious disease models is based on appropriate, detailed behavioural data. Although the self-report data collected by Mao and colleagues represents a good initial step towards incorporating behavioural data in infectious disease models, this data only assesses behavioural intentions rather than actual behaviour. Ideally, behavioural data should be observed directly within a target population during an infectious disease outbreak in order to ensure that the modelled behaviour is appropriate and relevant for the target population. This echoes a recommendation made in another recent review of the infectious disease modelling literature [21], thus underscoring the importance of using good behavioural data within this context.

### Limitations & further considerations

Despite the detailed, in-depth nature of our review, there are inevitably limitations and further considerations that need to be borne in mind while considering our results and recommendations. First, there were methodological limitations necessitated by time and resource constraints. While we did ensure that the search strategy was identical across all three electronic databases (using the HDAS search system), we did not optimise the thesaurus terms for each individual database. Furthermore, we did not conduct forward and backward citation searching of all included papers. Despite this, our search still revealed 1988 papers (excluding duplicates), with a total of 118 papers being subjected to the final full-text assessment (following the brief full text review occurring throughout our iterative screening process). We therefore believe that the scale and nature of our search and review was entirely appropriate given our emphasis on mapping and collating the extant literature rather than producing a full systematic assessment. In addition, we were not able to achieve full multiple-review of all of the papers retained for full-text analysis. There were, however, several iterations of our review strategy, and we did subject 50 papers that the first author was unsure over to review by multiple researchers. As mentioned previously, the resulting discussions over these 50 papers further contributed to the iterative development of our final inclusion/ exclusion criteria. Moreover, the primary reason for exclusion of papers at full-text stage is presented in Fig. 1 (the details of which individual papers were excluded for which reason are available from the first author on request).

Second, given the large number of relevant papers identified following the title/ abstract check it was necessary to concentrate our review; we chose to focus on endogenous, individual self-protective behaviour. By narrowly focusing our review, it is possible that we missed out on other interesting attempts to model human behaviour in the context of infectious disease spread. For example, our criteria precluded the inclusion of papers concerning the treatment of sexually transmitted disease and papers concerning the role of human behaviour in the spread of vector-borne diseases. Interestingly, our initial search strategy was designed to capture the full array of academic literature concerning the modelling of human behaviour in response to the spread of an infectious disease. It would therefore be possible for our dataset to be used to easily conduct reviews within these (and other) contexts in future.

Thirdly, as mentioned previously, there is a Western bias in the country of origin for the vast majority of all included papers. Given the cultural homogeneity in our sample, it is important to be aware of the impact that potential cultural differences in responses to emergency situations might have on the development of an infectious disease model. In much the same way as social group membership might impact upon behaviour during an infectious disease outbreak, other research has suggested that there may be ethnic or cultural differences in willingness to engage in health-related behaviours. For instance, research by Daphna Oyserman and colleagues found that racial-ethnic minority participants saw healthy behaviour (e.g., healthy eating) as behaviour that middle-class White individuals (and not themselves) engage in [84]. It is, therefore important for future modelling work to carefully consider the potential influence of both social and cultural influences on human behaviour in the aftermath of an infectious disease outbreak.

Finally, we are aware of two further reviews which together examine the incorporation of human behaviour within infectious disease models over the same time period as the current review [20, 21]. Although there is some overlap in the data extracted and conclusions drawn (particularly concerning the importance of behavioural data for parameterising models [21]), our review approaches the issues from an alternative perspective. Our primary emphasis is not on the precise mechanisms involved in infectious disease modelling, but is instead on contextualising the models within the extant psychological literature to provide recommendation for how this literature might be incorporated into future modelling efforts. By focusing more generally on the behavioural constructs that are currently modelled across the literature and how these relate to the psychological literature, our review presents a complementary analysis of the behavioural modelling literature from an explicitly psychological perspective. Given these differences, we therefore recommend that infectious disease modellers who are interested in incorporating human behaviour into their models should draw on all available reviews when attempting to develop future models incorporating human behaviour.

## Conclusions

Our scoping review of the infectious disease modelling literature identified 42 papers in which endogenous, individual self-protective behaviour is modelled. By extracting data including: the type of model, behaviour, and disease presented; the methods and constructs used to model self-protective behaviour, and; the theoretical basis for incorporating human behaviour in these models, we were able to develop a clear understanding of the ‘state of the art’ regarding the incorporation of human behaviour into infectious disease models.

By synthesising the key outcomes of this review with the extensive psychological literature concerning health behaviour prediction/change, we were able to make three key recommendations to help inform the modelling of infection prevention behaviour. First, modellers should consult established health behaviour change/ prediction theories to identify crucial, yet under-modelled behavioural constructs when developing their infectious disease models. We recommend the review of 83 theoretical models conducted by Susan Michie and colleagues [12] as a starting point for modellers to familiarise themselves with the range of available theories and constructs. Second, further stratification of social groups is recommended to improve complexity of social interaction and social influence within infectious disease models. Specifically, we recommend that modellers consult ‘The Social Cure’ literature (e.g., [15]) as an appropriate starting point. Finally, we recommend that modellers should use detailed, context-specific behavioural data (e.g., survey data, epidemiological vaccine uptake data, census data), wherever possible, to inform the development of their models.

## Additional files

Additional file 1: This file contains the Medline search strategy, the extraction criteria used, and an included papers reference list. (DOCX 25 kb)

Additional file 2: This excel spreadsheet contains the table detailing all extracted data summarised within the manuscript. (XLSX 26 kb)

## Abbreviations

EMBASE: Excerpta medical database
HDAS: Healthcare database advanced search
MEDLINE: Medical literature analysis and retrieval system online
MMR: Measles mumps and rubella
PICOS: Participants, interventions, comparisons, outcomes, and study design
PRISMA: Preferred reporting items for systematic reviews and meta-analyses
SIR: Susceptible-infected-recovered

## Footnotes

1 These terms were optimised for use in MEDLINE but were applied across the subsequent two searches. The only modification made to the PubMed search was to replace the ‘adj’ adjacency operators with ‘AND’. This was done as PubMed does not support the former type of operator. Furthermore the use of AND rather than adjacency operators would only have increased the potential number of papers identified through the search strategy.

2 The final two papers concerned facemask use [44] and sexual behaviour [36].

3 As each paper could contain more than one type of literature, they may be graphically represented in more than one bar.
