# Supplementary material for: Infection prevention behaviour and infectious disease modelling: a review of the literature and recommendations for the future
Source: BMC Public Health. 2018 Mar 9;18:336. doi: 10.1186/s12889-018-5223-1 (PMC5845221; doi:10.1186/s12889-018-5223-1)
Supplement: Supplementary file 1 — This file contains the Medline search strategy, the extraction criteria used, and an included papers reference list. (DOCX 25 kb) [file 12889_2018_5223_MOESM1_ESM.docx]

**Additional file 1**

**Medline Search Strategy**

2. Medline; behavio*.ti,ab; 829344 results.

3. Medline; BEHAVIOR/; 26977 results.

4. Medline; BEHAVIOR AND BEHAVIOR MECHANISMS/; 0 results.

5. Medline; BEHAVIORAL RESEARCH/; 2427 results.

6. Medline; HEALTH BEHAVIOR/; 35794 results.

7. Medline; PATIENT ACCEPTANCE OF HEALTH CARE/; 32289 results.

8. Medline; Communicable-disease*.ti,ab; 6329 results.

9. Medline; Infectious-disease*.ti,ab; 55396 results.

10. Medline; transmissable-disease*.ti,ab; 6 results.

11. Medline; (disease* adj3 transmiss*).ti,ab; 11471 results.

12. Medline; DISEASE TRANSMISSION, INFECTIOUS/; 6242 results.

13. Medline; COMMUNICABLE DISEASES/; 18013 results.

14. Medline; (theoretic* adj3 model*).ti,ab; 21469 results.

15. Medline; (mathematic* adj3 model*).ti,ab; 38369 results.

16. Medline; (epidemi* adj3 model*).ti,ab; 4478 results.

17. Medline; (econom* adj3 model*).ti,ab; 4191 results.

18. Medline; (comput* adj3 model*).ti,ab; 34700 results.

19. Medline; (first-order adj3 model*).ti,ab; 3955 results.

20. Medline; (hierarchical adj3 model*).ti,ab; 7115 results.

21. Medline; (time-series adj3 model*).ti,ab; 1561 results.

22. Medline; ARIMA.ti,ab; 562 results.

23. Medline; "Autoregressive Integrated Moving Average".ti,ab; 340 results.

24. Medline; static-decision.ti,ab; 12 results.

25. Medline; Markov.ti,ab; 13379 results.

26. Medline; Markov-chain.ti,ab; 3509 results.

27. Medline; Monte-carlo.ti,ab; 32012 results.

28. Medline; (Monte-Carlo adj3 model*).ti,ab; 3443 results.

29. Medline; "individual sample model*".ti,ab; 0 results.

30. Medline; (individual-sample* adj3 model*).ti,ab; 14 results.

31. Medline; (discrete-event adj3 model*).ti,ab; 297 results.

32. Medline; (discrete-event* adj3 simulat*).ti,ab; 455 results.

33. Medline; (discrete-event* adj3 simulat*).ti,ab; 455 results.

34. Medline; system-dynamic.ti,ab; 163 results.

35. Medline; (system* adj3 dynamic adj3 model*).ti,ab; 563 results.

36. Medline; stochastic*.ti,ab; 26551 results.

37. Medline; (stochastic* adj3 model*).ti,ab; 7105 results.

38. Medline; (dynamic-transmission adj3 model*).ti,ab; 77 results.

39. Medline; (mechanis* adj3 model*).ti,ab; 19972 results.

40. Medline; "mechan* model*".ti,ab; 5231 results.

41. Medline; (population-dynamic adj3 model*).ti,ab; 98 results.

42. Medline; (deterministic adj3 model*).ti,ab; 2225 results.

43. Medline; (compartment* adj3 model*).ti,ab; 16491 results.

44. Medline; (static adj3 model*).ti,ab; 1662 results.

45. Medline; (reproducti* adj3 number*).ti,ab; 2496 results.

46. Medline; (reproducti* adj3 value*).ti,ab; 728 results.

47. Medline; "agent based model*".ti,ab; 700 results.

47. Medline; "agent based model*".ti,ab; 700 results.

48. Medline; ABM.ti,ab; 800 results.

49. Medline; (ABM AND model*).ti,ab; 196 results.

50. Medline; ("multi agent" adj3 model*).ti,ab; 54 results.

51. Medline; (susceptible adj3 infect* adj3 recover*).ti,ab; 288 results.

52. Medline; (susceptible adj3 infect* adj3 susceptible).ti,ab; 11242 results.

53. Medline; (susceptible adj3 infect* AND susceptible).ti,ab; 11242 results.

54. Medline; "susceptible infect* susceptible".ti,ab; 122 results.

55. Medline; SIR.ti,ab; 9900 results.

56. Medline; SIS.ti,ab; 3048 results.

57. Medline; (differen*-equation* adj3 model*).ti,ab; 1717 results.

58. Medline; (hybrid*adj3 AND model*).ti,ab; 0 results.

59. Medline; simulat*.ti,ab; 328711 results.

60. Medline; (simulat* adj3 model*).ti,ab; 38439 results.

61. Medline; (spatial adj3 model*).ti,ab; 5389 results.

62. Medline; (chao* adj3 model*).ti,ab; 481 results.

63. Medline; network-analys*.ti,ab; 5585 results.

64. Medline; (network* adj3 analys*).ti,ab; 12814 results.

65. Medline; "additive mixed model*".ti,ab; 104 results.

66. Medline; (additive-mixed adj3 model*).ti,ab; 114 results.

67. Medline; ("realistic age" adj3 structur*).ti,ab; 18 results.

68. Medline; "realistic age structure model*".ti,ab; 1 results.

69. Medline; "realistic age structur*".ti,ab; 17 results.

70. Medline; "Nash equil*".ti,ab; 171 results.

71. Medline; "nash equilib*".ti,ab; 171 results.

72. Medline; "game theor*".ti,ab; 1103 results.

73. Medline; (bound* adj3 rational*).ti,ab; 151 results.

74. Medline; "social network*".ti,ab; 8950 results.

75. Medline; (social adj3 network*).ti,ab; 10781 results.

76. Medline; network-model*.ti,ab; 6498 results.

77. Medline; (network adj3 model*).ti,ab; 10951 results.

78. Medline; meta-model*.ti,ab; 104 results.

79. Medline; (meta adj3 model*).ti,ab; 2297 results.

80. Medline; (individual* adj3 based adj3 model*).ti,ab; 1508 results.

81. Medline; MODELS, THEORETICAL/; 112019 results.

82. Medline; "state model*".ti,ab; 3093 results.

83. Medline; (statistic* adj3 model*).ti,ab; 21040 results.

84. Medline; Statistic*-Model*.ti,ab; 10276 results.

85. Medline; Operation*-Research-Model*.ti,ab; 18 results.

86. Medline; (Operation*-Research adj3 Model*).ti,ab; 40 results.

87. Medline; Engineering-Model*.ti,ab; 188 results.

88. Medline; Physical-Model*.ti,ab; 2436 results.

89. Medline; (General* adj5 Model*).ti,ab; 36602 results.

90. Medline; (Linear adj5 Model*).ti,ab; 49995 results.

91. Medline; GAMM.ti,ab; 58 results.

92. Medline; GAM.ti,ab; 768 results.

93. Medline; GLM.ti,ab; 1470 results.

94. Medline; GLMM.ti,ab; 237 results.

95. Medline; Mixed-Effect-Model*.ti,ab; 1255 results.

96. Medline; (Mixed adj3 Model*).ti,ab; 20315 results.

97. Medline; Zero-Inflated-Model*.ti,ab; 52 results.

98. Medline; Hurdle-Model*.ti,ab; 96 results.

99. Medline; Regression-Model*.ti,ab; 81114 results.

100. Medline; Delay-Equation*.ti,ab; 55 results.

101. Medline; (Delay-Equation adj3 Model*).ti,ab; 4 results.

103. Medline; ANIMALS/ NOT HUMANS/; 3929175 results.

104. Medline; contagio*.ti,ab; 8792 results.

105. Medline; (disease* adj3 communic*).ti,ab; 7686 results.

106. Medline; (Infecti* adj3 disease*).ti,ab; 94258 results.

107. Medline; (Transmi* adj3 disease*).ti,ab; 27728 results.

112. Medline; (hybrid* adj3 model*).ti,ab; 3134 results.

113. Medline; "individual sampl* model*".ti,ab; 7 results.

114. Medline; (individual-sampl* adj3 model*).ti,ab; 23 results.

116. Medline; 2 OR 3 OR 4 OR 5 OR 6 OR 7; 887588 results.

120. Medline; (Bayes* adj3 Model*).ti,ab; 6576 results.

121. Medline; (Regression adj3 Model*).ti,ab; 91731 results.

122. Medline; (Hurdle adj3 Model*).ti,ab; 142 results.

123. Medline; (Zero-Inflated adj3 Model*).ti,ab; 350 results.

124. Medline; (Physical adj3 Model*).ti,ab; 5939 results.

125. Medline; (Engineering adj3 Model*).ti,ab; 1119 results.

126. Medline; (state adj3 model*).ti,ab; 11185 results.

127. Medline; (agent-based adj3 model*).ti,ab; 872 results.

128. Medline; (agent adj3 based adj3 model*).ti,ab; 910 results.

132. Medline; COMMUNICABLE DISEASE CONTROL/; 18356 results.

133. Medline; COMPUTING METHODOLOGIES/; 979 results.

134. Medline; 8 OR 9 OR 10 OR 11 OR 12 OR 13 OR 104 OR 105 OR 106 OR 107 OR 132; 159771 results.

135. Medline; 14 OR 15 OR 16 OR 17 OR 18 OR 19 OR 20 OR 21 OR 22 OR 23 OR 24 OR 25 OR 26 OR 27 OR 28 OR 29 OR 30 OR 31 OR 32 OR 33 OR 34 OR 35 OR 36 OR 37 OR 38 OR 39 OR 40 OR 41 OR 42 OR 43 OR 44 OR 45 OR 46 OR 47 OR 47 OR 48 OR 49 OR 50 OR 51 OR 52 OR 53 OR 54 OR 55 OR 56 OR 57 OR 58 OR 59 OR 60 OR 61 OR 62 OR 63 OR 64 OR 65 OR 66 OR 67 OR 68 OR 69 OR 70 OR 71 OR 72 OR 73 OR 74 OR 75 OR 76 OR 77 OR 78 OR 79 OR 80 OR 81 OR 82 OR 83 OR 84 OR 85 OR 86 OR 87 OR 88 OR 89 OR 90 OR 91 OR 92 OR 93 OR 94 OR 95 OR 96 OR 97 OR 98 OR 99 OR 100 OR 101 OR 112 OR 113 OR 114 OR 120 OR 121 OR 122 OR 123 OR 124 OR 125 OR 126 OR 127 OR 128 OR 133; 780827 results.

138. Medline; (Differen* adj3 equation*).ti,ab; 10462 results.

139. Medline; (Delay-Equation* adj3 Model*).ti,ab; 9 results.

140. Medline; 135 OR 138 OR 139; 785330 results.

141. Medline; 116 AND 134 AND 140; 1104 results.

142. Medline; 141 NOT 103; 996 results.

**Extraction criteria**

| 1. Authors | 2. Date | 3. Type of model | 4.  What behaviour is included? | 5.  How it is modelled? | 6. Theoretical Background (Y/N)? | 7.  If Y, what? | 8.  Is there a comparison to control model? | 9.  If Y, what are the results of the comparison? | 10.  Main conclusion (concerning behaviour) | 11.  Which disease is modelled? | 12.  Theoretical exercise or applied to data (if so, which data)? | 13.  Information on information/awareness spread | 14.  Information on fading/ decaying memory | 15. Country of origin |
| --- | --- | --- | --- | --- | --- | --- | --- | --- | --- | --- | --- | --- | --- | --- |

NB. Criteria 11-14 added following the interim presentation of review outcomes on 29^th^ February 2016. Criteria 15 added following Public Involvement workshop on 30^th^ September 2016

**Included papers reference list**

Andrews, A., & Bauch, T. (2015). Disease interventions can interfere with one another through disease-behaviour interactions. *PLoS Computational Biology, 11*(6), e1004291. doi: 10.1371/journal.pcbi.1004291

Auld, M. C. (2003). Choices, beliefs, and infectious disease dynamics. *Journal of Health Economics, 22*(3), 361-377. https://doi.org/10.1016/S0167-6296(02)00103-0

Barrett, C., Bisset, K., Leidig, J., Marathe, A., & Marathe, M. (2011). Economic and social impact of influenza mitigation strategies by demographic class. *Epidemics, 3*(1), 19-31. doi: 10.1016/j.epidem.2010.11.002

Bhattacharyya, S., & Bauch, C. T. (2010). A game dynamic model for delayer strategies in vaccinating behaviour for pediatric infectious diseases. *Journal of Theoretical Biology, 267*(3), 276-282. doi:10.1016/j.jtbi.2010.09.005

Bhattacharyya, S., & Bauch, T. (2011). "Wait and see" vaccinating behaviour during a pandemic: a game theoretic analysis. *Vaccine, 29*(33), 5519-5525. https://doi.org/10.1016/j.vaccine.2011.05.028

Chen, F. (2012). A mathematical analysis of public avoidance behavior during epidemics using game theory. *Journal of Theoretical Biology, 302*, 18-28. https://doi.org/10.1016/j.jtbi.2012.03.002

Chen, F., Jiang, M., Rabidoux, S., & Robinson, S. (2011). Public avoidance and epidemics: insights from an economic model. *Journal of Theoretical Biology, 278*(1), 107-119. https://doi.org/10.1016/j.jtbi.2011.03.007

Chen, F. H. (2006). A susceptible-infected epidemic model with voluntary vaccinations. *Journal of Mathematical Biology, 53*(2), 253-272. doi: 10.1007/s00285-006-0006-1

Chen, F. H. (2006). On the transmission of HIV with self-protective behavior and preferred mixing. *Mathematical Biosciences, 199*, 141-159. doi:10.1016/j.mbs.2005.12.004

Chen, F. H. (2009). Modeling the effect of information quality on risk behavior change and the transmission of infectious diseases. *Mathematical Biosciences, 217*, 125-133. doi:10.1016/j.mbs.2008.11.005

Cornforth, M., Reluga, C., Shim, E., Bauch, T., Galvani, P., & Meyers, L. A. (2011). Erratic flu vaccination emerges from short-sighted behavior in contact networks. *PLoS Computational Biology, 7*(1), e1001062. doi:10.1371/journal.pcbi.1001062

d'Onofrio, A., Manfredi, P., & Poletti, P. (2012). The interplay of public intervention and private choices in determining the outcome of vaccination programmes. *PLoS ONE, 7*(10), e45653. doi:10.1371/journal.pone.0045653.

Durham, P., & Casman, A. (2012). Incorporating individual health-protective decisions into disease transmission models: a mathematical framework. *Journal of the Royal Society Interface, 9*(68). doi: 10.1098/rsif.2011.0325

Fenichel, E. P. (2013). Economic considerations for social distancing and behavioral based policies during an epidemic. *Journal of Health Economics, 32*, 440-451. doi:10.1016/j.jhealeco.2013.01.002

Fenichel, E. P., Castillo-Chavez, C., Ceddia, M. G., Chowell, G., Parra, P. A. G., Hickling, G. J., . . . Villalobos, C. (2011). Adaptive human behavior in epidemiological models. *Proceedings of the National Academy of Sciences of the United States of America, 108*, 6306-6311. doi:10.1073/pnas.1011250108

Fu, F., Rosenbloom, D. I., Wang, L., & Nowak, M. A. (2011). Imitation dynamics of vaccination behaviour on social networks. *Proceedings of the Royal Society B Biological Sciences, 278*(1702), 42-49. doi: 10.1098/rspb.2010.1107

Funk, S., Gilad, E., Watkins, C., Jansen, V. A. A. (2009). The spread of awareness and its impact on epidemic outbreaks. *Proceedings of the National Academy of Sciences of the United States of America, 106*(16), 6872-6877. doi: 10.1073/pnas.0810762106

Guo, D., Li, K. C., Peters, T. R., Snively, B. M., Poehling, K. A., & Zhou, X. (2015). Multi-scale modeling for the transmission of influenza and the evaluation of interventions toward it. *Scientific Reports, 5*. doi: 10.1038/srep08980

Hayashi, M. A., & Eisenberg, M. C. (2015). Effects of adaptive protective behavior on the dynamics of sexually transmitted infections. *Journal of Theoretical Biology, 388*(7), 119-130. doi:10.1016/j.jtbi.2015.08.022

Karimi, E., Schmitt, K., & Akgunduz, A. (2015). Effect of individual protective behaviors on influenza transmission: an agent-based model. *Health Care Management Science, 18*(3), 318-333. doi: 10.1007/s10729-014-9310-2.

Liu, F., Enanoria, W. T. A., Zipprich, J., Blumberg, S., Harriman, K., Ackley, S. F., … Porco, T. C. (2015). The role of vaccination coverage, individuals behaviors, and the public health response in the control of measles epidemics: an agent based simulation for California. *BMC Public Health, 15*. doi: 10.1186/s12889-015-1766-6

Liu, X-T., Wu, Z-X., & Zhang, L. (2012). Impact of committed individuals on vaccination behaviour. Physical Review E, 86, 051132. doi: 10.1103/PhysRevE.86.051132

Mao, L. (2011). Evaluating the combined effectiveness of influenza control strategies and human preventive behavior. *PLoS ONE, 6*(10), e24706. doi: 10.1371/journal.pone.0024706

Mao, L., & Yang, Y. (2012). Coupling infectious diseases, human preventive behavior, and networks – a conceptual framework for epidemic modeling. *Social Science & Medicine, 74*, 167-175. doi: 10.1016/j.socscimed.2011.10.012

Meloni, S., Perra, N., Arenas, A., Gómez, S., Moreno, Y., & Vespignani, A. (2011). Modeling human mobility responses to the large-scale spreading of infectious diseases. *Scientific Reports, 1*. doi: 10.1038/srep00062

Morsky, B., & Bauch, T. (2012). Outcome inelasticity and outcome variability in behaviour-incidence models: an example from an SEIR infection on a dynamic network. *Computational and Mathematical Methods in Medicine, 2012*, 652562. doi:10.1155/2012/652562

Ndeffo Mbah, M. L., Liu, J., Bauch, C. T., Tekel, Y. I., Medlock, J., Meyers, L. A., & Galvani, A. P. (2012). The impact of imitation on vaccination behavior in social contact networks. *PLoS Computational Biology, 8*(4), e1002469. doi: 10.1371/journal.pcbi.1002469

Nicolaides, C., Cueto-Felgueroso, L., & Juanes, R. (2013). The price of anarchy in mobility-driven contagion dynamics. *Journal of the Royal Society Interface, 10*(87). doi: 10.1098/rsif.2013.0495

Oraby, T., & Bauch, C. T. (2015). Bounded rationality alters the dynamics of paediatric immunization acceptance. *Scientific Reports, 5*. doi: 10.1038/srep10724

Oraby, T., Thampi, V., & Bauch, C. T. (2014). The influence of social norms on the dynamics of vaccinating behaviour for paediatric infectious diseases. *Proceedings of the Royal Society B, 281*, 20133172, http://dx.doi.org/10.1098/rspb.2013.3172

Perisic, A., & Bauch, C. T. (2009a). A simulation analysis to characterize the dynamics of vaccinating behaviour on contact networks, *BMC Infectious Diseases, 9*(77). doi: 10.1186/1471-2334-9-77

Perisic, A., & Bauch, C. T. (2009b). Social contact networks and disease eradicability under voluntary vaccination. *PLoS Computational Biology, 5*(2), e1000280. doi: 10.1371/journal.pcbi.1000280

Poletti, P., Ajelli, M., & Merler, S. (2011). The effect of risk perception on the 2009 H1N1 pandemic influenza dynamics. *PLoS ONE, 6*(2), e16460. doi: 10.1371/journal.pone.0016460

Poletti, P., Ajelli, M., & Merler, S. (2012). Risk perception and effectiveness of uncoordinated behavioral responses in an emerging epidemic. *Mathematical Biosciences, 238*(2), 80-89. http://dx.doi.org/10.1016/j.mbs.2012.04.003

Poletti, P., Caprile, B., Ajelli, M., Pugliese, A., & Merler, S. (2009). Spontaneous behavioural changes in response to epidemics. *Journal of Theoretical Biology, 260*(1), 31-40. https://doi.org/10.1016/j.jtbi.2009.04.029

Reluga, T. C. (2010). Game theory of social distancing in response to an epidemic. *PLoS Computational Biology, 6*(5), e1000793. doi:10.1371/journal.pcbi.1000793

Reluga, T. C., Bauch, C. T., & Galvani, A. P. (2006). Evolving public perceptions and stability in vaccine uptake. *Mathematical Bioscience, 204*(2), 185-198. doi: 10.1016/j.mbs.2006.08.015

Reluga, T. C., & Li, J. (2013). Games of age-dependent prevention of chronic infections by social distancing, *Journal of Mathematical Biology, 66*, 1527-1553. doi: 10.1007/s00285-012-0543-8

Tanaka, M. M., Kumm, J., & Feldman, M. W. (2002).Coevolution of pathogens and cultural practices: a new look at behavioral heterogeneity in epidemics. *Theoretical Population Biology, 62*, 111-119. doi: 10.1006/tpbi.2002.1585

Wells, C. R., & Bauch, C. T. (2012). The impact of personal experiences with infection and vaccination on behaviour-incidence dynamics of seasonal influenza. *Epidemics, 4*(3), 139-151. doi: 10.1016/j.epidem.2012.06.002

Wells, R., Tchuenche, M., Meyers, L. A., Galvani, P., & Bauch, T. (2011). Impact of imitation processes on the effectiveness of ring vaccination. *Bulletin of Mathematical Biology, 73*(11), 2748-2772. doi: 10.1007/s11538-011-9646-4

Zhang, H.-F., Yang, Z., Wu, Z.-X., Wang, B.-H., & Zhou, T. (2013). Braess's paradox in epidemic game: better condition results in less payoff. *Scientific Reports, 3*. doi: 10.1038/srep03292
